# Withdrawal rates as a consequence of disclosure of risk associated with manipulation of the cervical spine

**DOI:** 10.1186/1746-1340-18-27

**Published:** 2010-10-26

**Authors:** Jennifer M Langworthy, Lianne Forrest

**Affiliations:** 1 Anglo-European College of Chiropractic, 13-15 Parkwood Road, Bournemouth BH5 2DF, UK

## Abstract

**Background:**

The risk associated with cervical manipulation is controversial. Research in this area is widely variable but as yet the risk is not easily quantifiable. This presents a problem when informing the patient of risks when seeking consent and information may be withheld due to the fear of patient withdrawal from care. As yet, there is a lack of research into the frequency of risk disclosure and consequent withdrawal from manipulative treatment as a result. This study seeks to investigate the reality of this and to obtain insight into the attitudes of chiropractors towards informed consent and disclosure.

**Methods:**

Questionnaires were posted to 200 UK chiropractors randomly selected from the register of the General Chiropractic Council.

**Results:**

A response rate of 46% (n = 92) was achieved. Thirty-three per cent (n = 30) respondents were female and the mean number of years in practice was 10. Eighty-eight per cent considered explanation of the risks associated with any recommended treatment important when obtaining informed consent. However, only 45% indicated they always discuss this with patients in need of cervical manipulation. When asked whether they believed discussing the possibility of a serious adverse reaction to cervical manipulation could increase patient anxiety to the extent there was a strong possibility the patient would refuse treatment, 46% said they believed this could happen. Nonetheless, 80% said they believed they had a moral/ethical obligation to disclose risk associated with cervical manipulation despite these concerns. The estimated number of withdrawals throughout respondents' time in practice was estimated at 1 patient withdrawal for every 2 years in practice.

**Conclusion:**

The withdrawal rate from cervical manipulation as a direct consequence of the disclosure of associated serious risks appears unfounded. However, notwithstanding legal obligations, reluctance to disclose risk due to fear of increasing patient anxiety still remains, despite acknowledgement of moral and ethical responsibility.

## Introduction

Autonomy is a concept that has received increased emphasis in health care in recent years [1]. Personal autonomy can be defined as self-determination that is not affected by either the controlling interference of others, or limitations, such as impeded comprehension [2]. To respect their autonomy, the clinician must acknowledge the patient's right to make decisions based on their individual views, values and beliefs and realise that a patient cannot make an autonomous decision unless they are well informed [2,3]. Nevertheless, it would appear that there is little agreement as to the parameters of autonomy and the limits of its validity [2,4].

Recognition and respect for the patient's right to autonomy is fundamental to ethical clinical practice and this is recognised in British Law [4-6]. Furthermore, it has been suggested that patients who actively exercise their autonomy with regard to their health care improve faster and more surely than those who do not [5]. Patients exercise their autonomy when choosing to see a doctor of their choice, be it the general practitioner (GP), chiropractor, osteopath or any other practitioner. Yet the most significant threat to their autonomy comes from the very practitioner they choose to see, as the clinician's specialist knowledge and training often inhibits patient authority [7]. Within the chiropractic profession, recent studies seem to support this view and suggest that when seeking chiropractic care, a patient's autonomy and right to self-determination may often be compromised [6].

Informed consent is an important part of the examination and treatment processes for all healthcare professionals, both legally and ethically. Legally it is an acceptable form of risk management and failure to obtain valid consent is an example of unacceptable professional practice which, within the UK chiropractic profession, contravenes the Code of Practice and Standard of Proficiency [8] laid down by its statutory regulatory body, the General Chiropractic Council (GCC). Complaints arising from non-compliance with the Code and Standard could, if upheld, amount to unacceptable professional conduct and lead to either admonishment, the issuance of a Conditions of Practice order, suspension or removal of the practitioner from the register. Such complaints could also result in civil charges of negligence or malpractice. Ethically it is grounds for the promotion of patient autonomy [9-11]. It is a continuous process during which the patient is provided with all pertinent information regarding proposed clinical procedures. It should include disclosure of associated risks and benefits, as well as alternative treatment options if the patient is to make an informed decision to proceed or otherwise with treatment.

The guidance [8] provided by the GCC states that patients have a right to accurate, relevant and clear information about the care available to them, inclusive of foreseeable risk, and that the provision of such information and the patient's understanding thereof is of greater importance than how they give consent and how it is recorded. Meanwhile, the UK's General Medical Council (GMC) guidelines [12] are a little less equivocal and highlight the practitioner's duty to provide patients with "clear, accurate information about the risks of any proposed investigation or treatment .... even if the likelihood [of a serious adverse outcome] is very small". Moreover, each condition has its own inherent complications quite apart from the selected therapy. Therefore, consent needs to be sought for every condition and therapy and not simply per patient. It is also important that the patient understands that in the process they are being asked to give consent to the procedures described. Thus the consent process provides an opportunity for the patient to exercise autonomy by participating in the decision-making affecting their health and care and allowing them to exercise their right to govern what happens to their body [13].

The literature suggests that inconsistency, non-compliance and/or a poor understanding of valid consent processes exists within chiropractic practice [14]. However, while formal consent is often not obtained, chiropractors have been found to be thorough in explaining procedures. Conversely, it has also been shown that they are reluctant to discuss possible serious adverse effects of treatment [6,9,14]. Reasons for this include the belief that the risk is unproven and minimal and that to inform patients of the possibility of harm, however small, will only serve to cause alarm [6,14]. Where risk remains unproven, it has also been suggested that practitioners may be uncomfortable revealing uncertainty and feel that doubt may suggest weakness of the profession [2].

In common with other forms of health care, chiropractic treatment carries with it an element of risk, the most controversial and well known being a purported association between cerebrovascular accident (CVA), or stroke, and manipulation of the cervical spine, which can ultimately result in permanent neurological damage or, in extreme cases, death [15]. Due to the rarity of CVA following cervical spine manipulation, accurate quantification of its risk has proven elusive and its reported frequency is somewhat ambiguous. According to Assendelft *et al* (1996) and Dvorak & Orelli (1985) respectively, as cited by Haldeman, Kohlbeck & McGregor (1999) [16], estimations range from a low incidence of 1 in 1.3 million manipulations to a high of 1 in 400,000. In a study investigating referral bias on the differences in perceived incidence of vertebral artery dissection (VAD) after cervical manipulation between neurologists and chiropractors, Haldeman, Carey, Townsend & Papadopoulos (2002) [17] estimated a rate of VAD dissection after manipulation of 1:5.8 million cervical manipulations. As cautioned by the authors, however, due to the nature of the study, this figure cannot be interpreted as representing the actual risk of stroke after manipulation. Others [18] have calculated the risk of any serious adverse reaction to cervical manipulation as being, at worst, 1 serious event in every 10,000 treatment consultations, although it should be noted that this rate of incidence is an estimate based on the rule of three [19]. Nonetheless, it is also thought that a number of these events may go unreported and thus the risk may be higher than commonly cited figures suggest [20].

The above illustrates how difficult it is to quantify the material risks [21] related to cervical manipulation. It highlights the need for further exploration of the causes and incidence of serious adverse reactions to the treatment to be able to promote or refute with sound evidence both its potential benefits and against unreasonable criticism [18,20]. Despite considerable investigation, the precise mechanism through which cervical manipulation is thought to produce such arterial damage is poorly understood [15]. It has been theorised that a minor trauma or simple self-induced head and neck movement could also have the same effect of artery dissection as a cervical manipulation if the individual is predisposed to this and has existing damage to the artery [22]. Currently, it is not possible to unequivocally determine who is at risk of experiencing such complications [15]. Despite the inability to determine an individual's risk of experiencing serious complications following cervical manipulation, it continues to be utilised in patient care as current evidence suggests the benefits outweigh the risks [23]. Nevertheless, it is imperative that the patient is fully aware of associated risks to treatment, as well as their magnitude, and understands them well in order to implement autonomy and provide valid informed consent based on their belief systems [11,13-16,18,20].

Within the chiropractic literature, it is evident that disclosure of serious risk associated with cervical manipulation remains controversial and poorly implemented and largely stems from a fear on the part of the chiropractor that to reveal the risk may alarm the patient and consequently lead to their withdrawal from care [6,14]. However, there is no evidence to either support or dismiss this fear. This study investigated withdrawal rates from treatment as a direct result of the disclosure of the risk associated with cervical manipulation.

## Methods

This study was reviewed by an internal Board for feasibility and ethics. Following approval, a questionnaire comprising 14 questions was developed and piloted on 5 currently practising chiropractors and feedback sought on clarity and relevance. Following minor amendment, a copy of the questionnaire, together with a covering letter and stamped return envelope, was sent to each of 200 randomly selected chiropractors registered with the UK General Chiropractic Council (GCC). Practitioners were selected using the computer random number generation facility within the Statistical Package for the Social Sciences (SPSS) v 16. The questionnaires were pre-coded prior to distribution to enable follow-up of non-responders.

Withdrawal rates were calculated as follows:

(i) Withdrawal rates over previous 12 months:

Number of patients for whom cervical manipulation was considered appropriate treatment but who reportedly withdrew from treatment as a direct consequence of the disclosure of serious risk, divided by the number of respondents who disclosed data in response to the question.

(ii) Withdrawal rate for total time in practice:

For each respondent who provided the data, the number of patients for whom cervical manipulation was considered appropriate but reportedly withdrew from treatment as a direct consequence of the disclosure of serious risk, divided by the individual respondents' number of years in practice. These numbers were then summed and divided by the number of respondents who disclosed data in response to the question.

All participants were assured of confidentiality and anonymity and no information was disclosed to any third party. Data were subjected to descriptive frequency analysis.

## Results

A response rate of 46% (n = 92) was achieved. Of those who responded, one-third (n = 30) were female. The majority (n = 21) were aged 36-40 years and respondents had a mean of 10 years practice experience. A little over one half (53%) (n = 49) graduated from the Anglo-European College of Chiropractic, 16% (n = 15) the Welsh Institute of Chiropractic and 11% (n = 10) from the McTimoney College of Chiropractic. The remaining 20% (n = 18) were graduates of other chiropractic institutions in the UK, US, Australia and South Africa.

Seventy-one per cent (n = 55) of respondents reported that 26-50% of patients presenting to their clinic in the preceding 12 months did so with neck pain. Cervical manipulation was considered appropriate treatment for 76-100% of neck pain patients by nearly two-thirds (63%) (n = 58) of the responding chiropractors.

Table 1 shows the elements of consent that respondents considered important. As shown, the majority (88%) (n = 81) considered an explanation of risk associated with recommended treatment important. However, when asked if they discuss this with patients in need of cervical manipulation, less than half (45%) (n = 41) reported that they always do so. Forty-one per cent (n = 38) indicated they sometimes discuss the issue, while 5% (n = 5) said they never do. For those patients requiring cervical manipulation, informed consent is obtained only for the first treatment by 71% (n = 65) of the responding chiropractors, compared to 15% (n = 14) who reported obtaining consent for each treatment. The remaining 13% (n = 12) said they obtain consent at other times during care, as shown below:

• Before first treatment

• Before first treatment and each thereafter until the patient is very familiar with the procedure

• With re-examinations

• New complaint and/or new course of care

• If there is a change to patients symptoms, or trauma

• As appropriate

• Written at first appointment and treatment, thoroughly verbally at report of findings and always verbally, i.e. "I'll adjust your neck now" to give them an opportunity to say no

• Ask if it's ok to go ahead at each treatment

• First treatment then when there are changes to treatment protocol

• Ongoing

• First treatment and every 12^th ^visit thereafter

• Initially with first visit and then periodically depending on symptoms

**Table 1:** Elements of Consent Considered Important in the Securing of Valid Informed Consent

	Yes (%)
Explanation of the examination process	79 (86)
Explanation of associated risks associated with recommended treatment	81 (88)
Explanation of the benefits of recommended treatment	84 (91)
Discussion of alternative treatment(s) and their risks and benefits	48 (54)

### Communicating Risk

Respondents were asked how they explain and quantify the risk associated with cervical manipulation to their patients. Approximately, one-third (37%) (n = 34) stated they quote figures on CVA risk from the literature. Figures cited ranged from 1:1000 to 1:12 million treatment visits. Assessment for known risk factors and an explanation of these were reportedly undertaken by 15% (n = 14) of the sample, while 9% (n = 8) reported giving their patients reading material outlining the risk. Nearly one-third (30%) (n = 28) stated they like to use comparisons to everyday hazards to put the risk in perspective. These include the risks associated with medication, specifically non-steroidal anti-inflammatory drugs (NSAIDS), aspirin and paracetamol, surgery and anaesthesia. Going to the hairdresser, driving or crossing the road and the chances of being struck by lightening or winning the lottery were also cited. Seven per cent (n = 6) said they only discuss risk if the patients themselves ask about it.

### Ethics and Disclosure

Respondents were asked whether they believed that discussing the possibility of a serious adverse reaction to cervical manipulation could increase patient anxiety to the extent that there was a strong possibility the patient would refuse treatment. Nearly half (46%) (n = 42) said they believed this was possible. A large majority (79%) (n = 73) also said they believed that as a chiropractor they had a moral and ethical obligation to disclose the risk associated with cervical manipulation. When asked if they believed this despite concerns it might lead to the patient refusing treatment, 80% (n = 74) said yes.

### Withdrawal Rates

Amongst patients for whom respondents considered cervical manipulation to be an appropriate treatment, there was found to be an estimated withdrawal rate of 18 patients for every 25 practitioners over the previous 12 month period as a direct consequence of the disclosure of the risk. Of the 75 respondents who provided withdrawal numbers, the majority (79%) (n = 73) reported no withdrawals in the preceding 12 months. The highest reported number of withdrawals was 27 for one respondent, with the remaining 16% (n = 15) reporting withdrawal numbers from 1-5 over the past 12 months. As the chiropractor who reported 27 withdrawals appeared atypical, the withdrawal rate was recalculated omitting this data. This produced an adjusted rate of 10 patients per 27 practitioners.

Respondents were also asked to estimate the number of patients who had withdrawn for the same reason throughout their total time in practice. This produced an estimated rate of 1 patient withdrawal for every 2 years in practice. Again, of those respondents who provided numbers (n = 76), almost half (46%) (n = 42) reported no withdrawals throughout their time in practice. The highest reported number of withdrawals was 100 over 13 years. Removal of this potential outlier had little effect and produced an estimated rate of 1 patient per 2.3 years of practice.

## Discussion

To extrapolate results beyond the participants of this study would require a minimum of 333 respondents from a total sampling of 666 subjects, based on a 50% response rate and a 95% level of confidence. As these criteria were not met, it is not appropriate to generalise the results, which carry an error level of 10%. Nonetheless, some important issues are raised which warrant further scrutiny amongst the wider chiropractic community. There is an opportunity for the profession to manage risk but only if it embraces it fully and takes ownership.

In recent years, patients have been increasingly encouraged to exercise their right to autonomy and to have a more active role in their own health care. To successfully do this, patients need to be cognisant of all pertinent issues arising from their complaint, diagnosis, prognosis and treatment, as well as the options available to them. By admission, many of the participants in this study often fail to fully comply with this which, in effect, undermines patient autonomy, invalidates the consent process and contravenes legal principles and professional codes of practice. Given the complexities of consent, some may feel this to be harsh and argue that the consent process as advocated in theory is somewhat divorced from the realities of daily practice. Nevertheless, complexity does not negate the responsibility of the practitioner to obtain valid consent.

Whilst undoubtedly there are some patients who present a greater challenge to the attainment of valid consent or who may be alarmed if made aware of any risk associated with treatment, it would seem that the number of patients to whom this applies and the levels of alarm experienced are perhaps exaggerated by the practitioner in an attempt to mask their own anxieties/insecurities in dealing with the more grey areas of clinical practice. In chiropractic and in relation to risk associated with cervical manipulation, this may be due to discomfort felt by the chiropractor in communicating the known but unproven threat of stroke. This is perhaps well illustrated by the large majority (80%) of respondents to the current study who believe they have a moral and ethical obligation to disclose the risk associated with cervical manipulation, despite concerns it might lead to patient withdrawal from treatment, yet less than half (45%) always do so. It would be advantageous for all if practitioners were willing to simply disclose what is currently known about the level of the risk and probability of occurrence, including that not all is yet known about it. This would be preferable to blaming the patient's unknown but assumed response to such information as reason for not disclosing the risk at all, or addressing it in a cursory and dismissive manner. When presented with potentially difficult choices, most individuals cope well provided they have been well informed and are given the right support [24]. Consequently, the often-made assumption that to inform patients of risk will only serve to increase anxiety and to the withdrawal from treatment rarely becomes a reality [25]. Yet for some practitioners it is extremely important to accentuate the "natural" or "holistic" image that chiropractic enjoys, at least subliminally (i.e. it is a 'natural' therapy and is therefore safe). To have to acknowledge and explain potential complications of the treatment may be felt by some to undermine this image.

Whilst respondents were asked to estimate withdrawal rates as a direct result of the disclosure of serious risk associated with cervical manipulation, one respondent commented that their patient withdrew as they found the procedure uncomfortable. As it is possible that other similar instances were inappropriately included in estimates, it is unknown if the estimates provided were all solely due to the disclosure of serious risk. Nor is it known whether patients withdrew completely from chiropractic care or continued to receive another modality of chiropractic care. Nevertheless, the withdrawal rates calculated for the preceding 12 month period and that for total time in practice were both low. A large majority indicated no withdrawals in the past year, while nearly half reported no withdrawals throughout their total time in practice. The aforementioned limitation in data collection may have had an effect on the withdrawal rate calculated. If only cases where patients withdrew completely from all chiropractic care were included, it is possible the resultant withdrawal rates may have been even lower. Moreover, if patient withdrawal numbers were to be calculated relative to the total number of patients seen per practitioner, the number of withdrawals would be proportionally lower again. This suggests that fears regarding the disclosure of risk causing increased patient anxiety leading to subsequent refusal of treatment may be largely unfounded.

Responses to a question on when consent is sought raised a few concerns. Predicting which patients are at risk of a serious adverse reaction to cervical manipulation is not an exact science, particularly since pre-manipulative testing for the detection of vascular insufficiency has been reported as having little clinical value [26]. In light of this, and given the devastating, possibly fatal, consequences of a CVA, it is difficult to see how a practitioner can differentiate with absolute certainty when it is and is not appropriate to seek consent. Similarly, the practitioner simply saying to the patient "I'll adjust your neck now" and expecting them to realise that permission is being sought to proceed, is unlikely to meet the standard of valid consent. Equally, the practice of seeking consent on the 1^st ^and every 12^th ^visit thereafter would not appear to be guided by each patient's particular circumstances and needs.

Of concern, a number of respondents in the current study reported only discussing risk if patients themselves raise the issue. Other studies [6] surveying chiropractic practice have found the same. This defies all ethical precepts of clinical practice and ignores the legal onus on practitioners to initiate the disclosure of all information that might reasonably be considered necessary to provide context for the patient to make an informed decision about treatment [27]. While what exactly is meant by 'reasonable' in this context may be arguable, to say nothing about the risk, be it established or known but unproven as in the case of cervical spine manipulation, is unacceptable at ethical, legal and professional levels. In a worst case scenario, it might also ultimately be judged as reckless and/or negligent. For the profession generally, as precedent demonstrates (e.g. UK law [28], if it is not seen to comply with or adequately enforce its code and standards of practice, the ultimate sanction could be that the privilege that is self-regulation is removed.

Finally, given the rarity of event and its elusiveness to quantification, some may argue it is inappropriate to apply the term 'risk' to the potential for a serious adverse reaction to cervical spine manipulation resulting in stroke or other significant neurological damage. Risk deemed to be material has been defined [21] as "a grave or detrimental consequence of treatment, regardless of the infrequency of its occurrence", while other sources [29,30] define it as 'the chance or possibility of loss or bad consequence' and as 'a person or thing causing a risk or regarded in relation to risk'. Whilst to date no unequivocal causal relationship between cervical manipulation and stroke has been established, the literature does seem to suggest a temporal association [22,31]. Current thought purports this might be due to patients presenting with headache and neck pain for manipulation with an already dissecting vertebrobasilar artery or with an inherent predilection [22,32,33]. Thus the effect of manipulation would not be one of causation but exacerbation. This theory may too prove controversial as a recent study [34] investigating the effect of cervical manipulation on a pre-existing lesion of the vertebral artery showed no significant difference in its length, area or volume pre- and post manipulation. This study was, however, limited to an animal model. Cassidy *et al*, (2009) [33] also found that patients presenting to a chiropractor were at 'no excess risk' of VBA [vertebrobasilar artery] stroke from chiropractic care than from that provided by a primary care physician. Nonetheless, no excess risk does not equate to no risk. Indeed, these authors do not rule out neck manipulation as a potential cause of some VBA strokes, albeit not a major one. According to current knowledge, whether temporal, causal or contributory, the possibility for a poor outcome appears to exist, thus constituting risk. Chiropractors must accept and disclose this to their patients in order to remain ethical, sensitive to patient autonomy and to retain credibility with external agencies.

## Conclusion

Results suggest that fears about increased patient anxiety leading to the withdrawal from care as a direct consequence of the disclosure of risk associated with cervical manipulation, may be unfounded. Inconsistency and non-compliance with the process of valid informed consent appears to remain a feature in some areas of UK chiropractic practice, despite acknowledgement of moral and ethical responsibility.

## Competing interests

The authors declare that they have no competing interests.

## Authors' contributions

JL conceived the study. LF undertook data collection and analysis. Both authors contributed to drafts and approved the final manuscript.
